# Identification of Compounds in the Essential Oil of Nutmeg Seeds (*Myristica fragrans* Houtt.) That Inhibit Locomotor Activity in Mice

**DOI:** 10.3390/ijms11114771

**Published:** 2010-11-23

**Authors:** Muchtaridi, Anas Subarnas, Anton Apriyantono, Resmi Mustarichie

**Affiliations:** 1 Faculty of Pharmacy, Universitas Padjadjaran, Jl KM 21.5 Bandung-Sumedang, Jatinangor, Indonesia; E-Mails: anassubarnas@unpad.ac.id (A.S.); resmi.mustarichie@unpad.ac.id (R.M.); 2 Department of Food Technology and Human Nutrition, Bogor Agricultural University, Jl. Darmaga, Bogor, Indonesia; E-Mail: apryan@indo.net.id

**Keywords:** nutmeg (*Myristica fragrans* Houtt.) seeds, essential oils, inhalation, GC-MS

## Abstract

The present study was designed to evaluate the inhibitory effect of nutmeg (*Myristica fragrans* Houtt.) seed essential oil on the locomotor activity of mice in a wheel cage. Active compounds in the essential oil were identified by off-line solid phase extraction (SPE-C18) and GC/MS analysis. The essential oil was administered by inhalation at doses of 0.1, 0.3, and 0.5 mL/cage. The results showed that inhalation of nutmeg seed essential oil at a dose of 0.5 mL/cage decreased locomotion by 68.62%; and inhalation of 0.1 and 0.3 mL/cage inhibited locomotion by 62.81% and 65.33%, respectively. Generally, larger doses and longer administrations of nutmeg seed essential oil exhibited greater locomotor inhibition. Subsequently, the plasma concentrations of essential oil compounds were measured. The most concentrated compound in the plasma was myristicin. Half an hour after the addition of 1 mL/cage of nutmeg seed oil, the plasma concentration of myristicin was 3.7 μg/mL; one and two hours after the addition, the blood levels of myristicin were 5.2 μg/mL and 7.1 μg/mL, respectively. Other essential oil compounds identified in plasma were safrole (two-hour inhalation: 1.28 μg/mL), 4-terpineol (half-hour inhalation: 1.49 μg/mL, one-hour inhalation: 2.95 μg/mL, two-hour inhalation: 6.28 μg/mL) and fatty esters. The concentrations of the essential oil compounds in the blood plasma were relatively low (μg/mL or ppm). In conclusion, the volatile compounds of nutmeg seed essential oil identified in the blood plasma may correlate with the locomotor-inhibiting properties of the oil when administered by inhalation.

## 1. Introduction

The ethnobotany of nutmeg (*Myristica fragrans* Houtt.) (MF, *Myristicaceae*) was studied in the provinces of Maluku and of central and east Java [1]. The utility of nutmeg as a spice has been known since ancient times in Indonesia, and nutmeg was probably introduced into Europe during the twelfth century. Nutmeg has aromatic, stimulant, narcotic, carminative, astringent, aphrodisiac, hypolipidaemic, antithrombotic, anti-platelet aggregation, antifungal, antidysenteric, and anti-inflammatory activities. The spice is used as a remedy for stomach ache, rheumatism, and vomiting during pregnancy [2,3]. Sonavanne *et al*. [3] explained that the n-hexane extract of nutmeg seeds has anxiogenic, sedative and analgesic activities. Nevertheless, no evidence was found to support the previously published reports of nutmeg’s hallucinogenic or other psychoactive properties besides mild sedation.

HPLC analyses of nutmeg seed essential oils have shown that alkylbenzes and arylproanoids predominate [4]. Behavioral studies have shown that the compounds have strong sedative effects [5]. The elevated plus maze is a widely used rodent model of anxiety in which decreased time spent in the open arm (increased time spent in the closed arm) suggests anxiogenic activity. Sonavanne *et al*. [6]. showed a significant decrease in the number of entries into both the open and the closed arms. Further, the authors documented a decrease in the ratio of entries into the open arm *versus* the number of entries into both arms, which suggests the anxiogenic activity of nutmeg. In addition, the effects of nutmeg seeds were dose-dependent. Thus, the observations of Sonavanne *et al*. [3] confirmed the anxiogenic effect of MF.

The toxicity of nutmeg extract was reported by Hallstrom and Thuvander [7] and Stein *et al*. [5]. Intoxication occurs in human after an ingestion of approximately 5 g of nutmeg, corresponding to 1–2 mg myristicin/kg body weight (b.w.) [7]. Acute intoxication with nutmeg causes gastrointestinal symptoms [5]. The main objective of this study was to identify the volatile, active compound in nutmeg seed essential oil that inhibits mouse locomotion.

## 2. Results and Discussion

### 2.1. Composition of Essential Oils

The essential oil of nutmeg seeds was isolated with a yield of 6.85% w/w. The major compounds in the oil were sabinene (21.38%), 4-terpineol (13.92%) and myristicin (13.57%). On the other hand, allylbenzene and propylbenzene derivatives (myristicin, safrole, eugenol, and derivatives thereof) were the predominant compounds in nutmeg seeds. The data describing all the compounds extracted from nutmeg seeds are presented in Table 1.

This research showed that the essential oil of nutmeg seeds is of good quality as it has ideal percentages of essential oil and active compounds. The standard of Materia Medica Indonesia (MMI) requires that the essential oil content in nutmeg seeds be 5 to 10%, and it was found to be 6.85% in this study. Additionally, the content of myristicin and safrole measured in this study exceeded MMI requirements of 5 to 10% [8].

### 2.2. Locomotor Activity of Mice after Inhalation of Nutmeg Oil

To determine the effects of nutmeg seed essential oil on locomotion, we administered it to mice via inhalation. A strong inhibitory effect of the essential oil on locomotion was observed; doses of 0.1, 0.3, and 0.5 mL/cage decreased locomotor activity by 62.81%, 65.33%, and 68.62%, respectively (Table 2). Thus, the effect of nutmeg oil on locomotion was dose-dependent.

**Figure 1.**
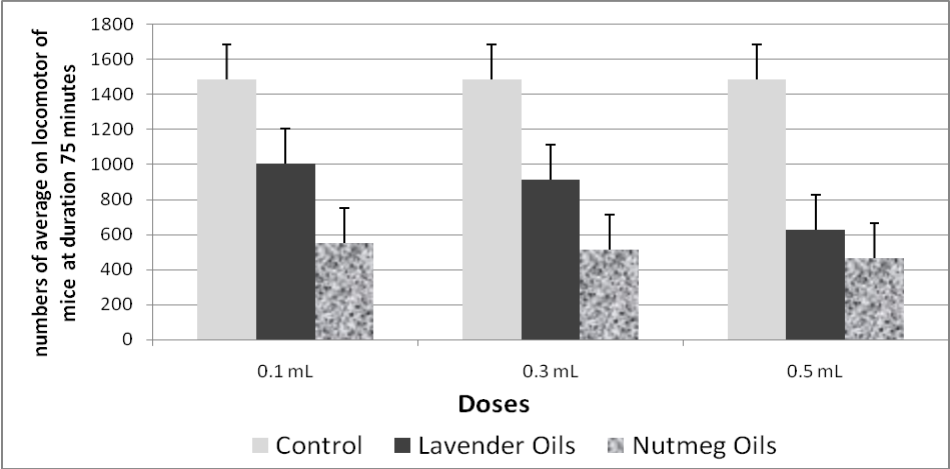
Bar group of the average numbers of locomotor activities of mice after 75 minutes inhalation of essential oils.

In this research, we compared the activity of nutmeg essence to that of lavender oil as positive control, because lavender oil has been proven to decrease locomotion of female and male laboratory animals [9]. As shown in Figure 1, nutmeg oil afforded a greater inhibitory effect than did lavender oil. Sonavanne *et al*. [6]. had previously shown that oral administration of nutmeg seeds reduces the number of head pokes and potentiates pentobarbital-induced sleep. The preceding observations suggest that nutmeg seeds have sedative effect. Since the mechanism of anxiogenic activity is obscure, it is difficult to infer the mechanism by which nutmeg seeds potentiate pentobarbital-induced sleep, but it is possible that the allybenzene or propylbenzene derivatives in nutmeg contribute to sedation.

### 2.3. Active Volatile Compounds

The roles of volatile compounds in locomotor activity can differ. However, all the active compounds in nutmeg seeds are contained in the essential oil. The compounds identified in blood plasma with high bioavailability after 30 to 120 minutes of exposure are likely responsible for the observed locomotor inhibition and are thus lead compounds [10].

**Figure 2.**
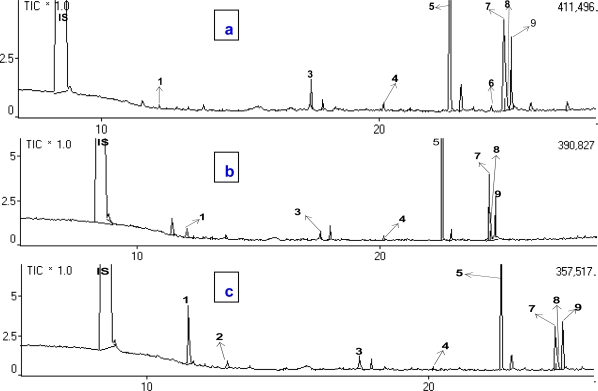
Total ion chromatogram of a blood plasma sample after inhalation of nutmeg oil. (**a**) chromatogram of the sample after a half-hour inhalation; (**b**) chromatogram of the sample after a one-hour inhalation; (**c**) chromatogram of the sample after a two-hour inhalation. **IS**: Internal standard (1,4-dichlorobenzene); **1**: 4-terpineol; **2**: safrole; **3**: myristicin; **4**: methyl myristate; **5**: methyl palmitate; **6**: palmitic acid; **7**: methyl 10-octadecanoate; **8**: methyl oleate; and **9**: methyl stearate.

In this study, myristicin and 4-terpineole were the predominant nutmeg seed essential oil compounds identified in mice made to inhale the extract. It was strange that sabinene, as the highest component in the nutmeg seeds essential oil, was not identified in the blood plasma. Furthermore, the concentrations of both myristicin and 4-terpineole in the blood plasma of mice increased with duration of exposure to nutmeg seed essential oil (Figure 2). Table 3 summarizes the volatile compounds found in blood plasma as a function of duration of inhalation.

On the other hand, some esters such as methyl myristate, methyl palmitate, methyl 10-octadecanate, methyl oleate, and methyl stearate were also identified in the plasma of mice following exposure to nutmeg seed essential oil. Of these esters, the plasma concentration of methyl palmitate exceeded that of the other compounds.

Half an hour after the addition of 1 mL/cage of nutmeg seed oil, the concentration of myristicin identified in the plasma was 3.7 μg/mL. After one and two hours inhalation of 1 mL of nutmeg seed oil, the myristicin levels in plasma increased to 5.2 μg/mL and 7.1 μg/mL, respectively (Table 3). Based on previous studies [6,11], myristicin, safrole, and 4-terpineol were hypothesized as the active compounds responsible for the inhibitory activity on the locomotion studied here. Structurally, safrole has the basic structure of an allylbenzene and of a propylbenzene, both of which contribute to the aromatic properties of plants.

Myristicin is a safrole derivative with methoxy group attached at carbon 4. The methoxy group in myristicin confers a strong sedative effect on the compound, as does a methoxy group in phenylpropanolamine [12]. Moreover, it has been reported that myristicin undergoes metabolism in the body, and its metabolite is known as a sedative 3-methoxy-4,5-methylenedioxyamphetamine (MMDA) [5,11]. This evidence supports the hypothesis that myristicin in nutmeg seed oil contributed to the locomotor inhibition in mice.

In this study, plasma levels of myristicin and safrole in mice correlated with extent of locomotor inhibition. After inhalation of nutmeg seed oil at a dose of 0.5 mL, mouse locomotor activity was inhibited by 68.82%. This effect was dose-dependent and more pronounced compared to that of other essential oils. Greater concentrations of myristicin were detected in plasma following inhalation of larger doses. Along the same lines, the concentration of 4-terpineol in blood plasma after inhalation of 0.1-, 0.3-, and 0.5-mL doses were 1.49, 2.95, and 6.28 μg/mL, respectively (Table 3).

Other authors [13,14] reported that 4-terpineol potentiates the GABA_A_ receptor-mediated response at a low concentration of GABA as effectively as α-terpineol. In particular, 4-terpineol potentiates the GABA response remarkably. The terpineols potentiate the effect of 5 μM GABA. The potentiation caused by a mixture of 4-terpineol and citral or cineol is greater than that caused by citral, cineol, or butanol, but less than that by 4-terpineol itself. This suggests there might be competitive binding of 4-terpineol and the three other compounds to the potentiating site.

### 2.4. Locomotor Activity of Single Compounds Identified in Mice

**Figure 3.**
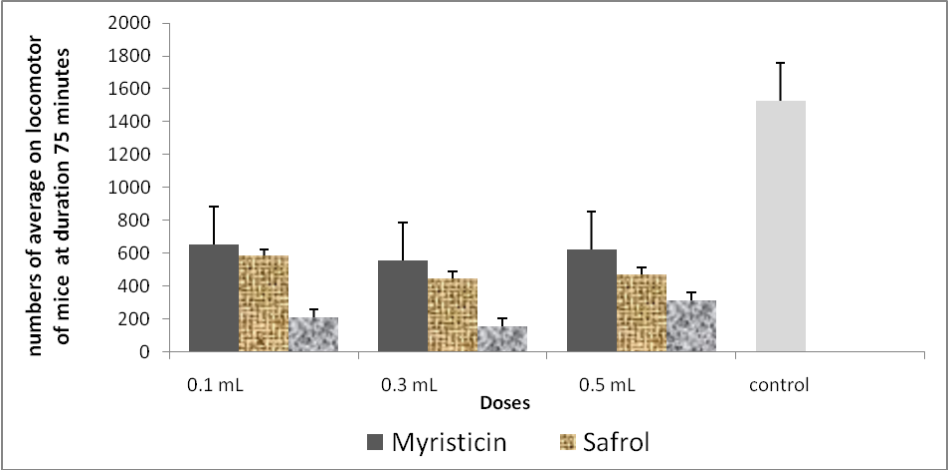
Graph groups of the average number of locomotor activities of mice after 75 minutes inhalation of myristicin, safrole, and 4-terpineol.

According to the results above, myristicin, safrole, and 4-terpineol verify our prediction that these three compounds play a role in inhibiting locomotor activity in mice. Figure 3 showed that myristicin, safrole, and 4-terpineole have a strong capacity to inhibit locomotor activity in mice. Safrole effects extreme inhibition compared to others as can be seen in Table 4.

Based on these results, we suppose that the volatile compounds detected in blood samples, such as myristicin, 4-terpineole, and safrole, were responsible for the observed inhibition of locomotor activity. It is suggested that locomotor inhibition by nutmeg seed essential oil is due, at least in part, to the direct pharmacological action of one or more of its constituents.

## 3. Experimental Section

### 3.1. Materials

**Animals**—Male mice weighing 25 to 30 g and 2 to 3 months old were used. The mice were adapted for one week to the laboratory in which locomotor activity experiments were conducted and were selected for wheel rotations of between 150 to 300 before the experiments were started.

**Aromatic plant material**—The nutmeg seeds (*Myristica fragrans* Houtt.) used were obtained from Bogor, West Java.

**Chemicals**—Methanol puriss. p.a. (Merck) was used as eluent for SPE. Heparin tubes (Boehringer) were used for blood collection. Pure lavender (*Lavandula officinalis*) oils were obtained from Martina Bertho. Alkane standards C_8_–C_20_, C_21_–C_40_, 1,4-dichlorobenzene, and myristicin, and safrole were obtained from Sigma.

### 3.2. Methods

#### 3.2.1. Isolation of Essential Oil

Dried nutmeg seeds (500 g) were water-distilled at Balitro, Monaco Lembang for 3 hours. The oil was stored at −20 °C after addition of sodium sulfate.

#### 3.2.2. Mouse Locomotor Activity Tests

Locomotor activity of mice was measured using a wheel cage, in which the mice ran and the number of rotations was recorded by a meter. Cage inhalators contained glass fiber (20 cm × 20 cm × 30 cm) and were equipped with an electric fan for the evaporation and distribution of volatile compounds. The mice were selected by weight (25 to 30 g) and by their ability to rotate the wheel cage up to 300 times in 30 minutes; eligible mice were then divided into three groups: a control group, a lavender oil positive control group (using 0.1, 0.3, and 0.5 mL/cage), and a nutmeg seed oil treatment group (using 0.1, 0.3, and 0.5 mL/cage). Each group consisting of 5 mice was tested three separate times. After 30 minutes of inhalation, the mice were placed into the wheel cage and after 5 minutes, the number of rotations was recorded for 75 minutes in 15-minute intervals.

#### 3.2.3. GC/MS Analysis

Measurements were performed using a QP-5050A (Shimadzu) gas chromatograph coupled to a VG Autospect Mass Spectrometer at 70 eV, 40–550 amu with a fused silica capillary column (DB-5MS, 30m × 0.25 mm) using helium as a carrier gas and with temperature programming from 60 °C/5 min to 300 °C/1 min (10 °C/ min) for blood plasma and 60 °C/5 min to 300 °C/2 min (10 °C/ min) for essential oils. The MS was operated using an interface temperature of 240 °C, and an electron impact ionisation of 70 eV with a scan mass range of 40–350 *m/z* (sampling rate of 1.0 scan/s).

#### 3.2.4. Qualitative Analysis

Identification of the compounds was conducted by comparing their linear retention indices (LRI) with literature values and their mass spectral data with those from the MS data system (Willey-229 lib., Nist-62 lib., and Nist-12 lib) [15]. Linear retention indices were calculated using GC data of a homologous series of saturated aliphatic hydrocarbons (C_8_ to C_40_) separated on the same column using the same conditions as for GC analysis of the essential oils and the blood samples. The blood samples were collected from the corner parts of the eyes using capillary tubes and placed in a heparin tube.

#### 3.2.5. Quantitative Analysis

Detailed analysis was performed using a modification of the methods of Jirovetz *et al*. [16,17] and Kovar *et al*. [18]. The blood samples (500 to 600 μL), obtained according to [17], were centrifuged (1800 rpm/10 min) at room temperature and concentrated on a C_18_-column (100 mg Sep-Pak, Waters). Volatile compounds were separated using a mobile phase of the mixture of methanol—bidistilled water (60:40). Five microlitees were injected into the GC-MS. Quantification of the volatile compounds in the blood samples was accomplished using 1,4-dichlorobenzene 0.5% (500 μL) as an internal standard according to the following equation:

$$[C]=\frac{A}{IS}\times \frac{IS\;\text{weight}\;(\text{g})}{100\;\text{mL}}\times \%EO\times IS\;\text{volume}\;\times 10^{6}$$

*C*: concentration (g/g);
*IS*: GC peak area of Internal Standard
*A*: GC peak area of compounds of essential oils
%*EO*: yield of essential oils.

## 4. Conclusions

This study reports that the volatile compounds detected in blood samples such as myristicin, 4-terpineole and safrole were associated with inhibition of locomotor activity in mice. It is suggested that the locomotor inhibition by nutmeg seed essential oil is due, at least in part, to the direct pharmacological action of one or more of its constituents.

## Figures and Tables

**Table 1.** Chemical composition of essential oil of nutmeg seeds (*Myristica fragrans* Houtt.).

**No.**	**Retention Time**	**LRI exp^a^**	**LRI Ref^b^**	**Compounds**	**Percentage %**
1.	5.967	920	931	α-thujene	0.78
2.	6.246	931	939	α-pinene	10.23
3.	6.583	943	953	Camphene	0.16
4.	7.508	978	976	Sabinene	21.38
5.	7.792	989	991	α-myrcena	2.38
6.	8.493	1017	1018	α-terpinene	2.72
7.	8.843	1032	1031	Limonene	5.57
8.	9.142	1045	1040	β-ocimene	0.03
9.	9.525	1061	1062	γ-terpinene	3.98
10.	9.728	1070	1097	*trans*-sabinene hydrate	0.03
11.	10.097	1085	1088	Terpinolene	1.62
12.	10.393	1098	1098	Linalool	0.75
13.	10.821	1119	-	Fenchyl alcohol	0.05
14.	11.603	1158	1219	*cis*-sabinene hydrate	0.06
15.	12.306	1193	1177	4-terpineol	13.92
16.	12.492	1203	1189	α-terpineol	3.11
17.	12.67	1212	1228	Citronellol	0.77
18.	13.297	1247	1257	Linalyl acetate	0.06
19.	13.949	1282	1285	Bornyl acetate	0.24
20.	14.158	1293	1285	Safrole	4.28
21.	15.76	1392	1401	Methyl eugeunol	0.77
22.	16.568	1447	1447	Isoeugeunol	1.74
23.	17.773	1530	1520	Myristicin	13.57
24.	18	1551	1554	Elimicin	1.42
25.	18.573	1586	-	Metoxyeugeunol	0.10
26.	18.742	1598	1622	β-asaron	0.03
27.	20.625	1767	-	Myristic acid	0.11
28.	21.01	1789	1793	Ethyl miristate	0.04
29.	21.352	1716	-	Palmitic acid	0.03
30.	22.946	1954	1993	Ethyl palmitate	0.07
31.	25.103	2181	-	Stearic acid	0.01
32.	25.183	2193	2194	Ethyl oleate	0.01

a: LRI reference in Adams (1995) with DB5 column;

b: LRI experiment with DB5-MS column.

**Table 2.** Average number of mice wheel cage rotations within 75 minutes of inhalation of nutmeg and lavender essential oils.

**No**	**Mean instead of Number of average**								**Inhibitory Effect^c^ (%)**
	**Treatment**	**Doses (mL)**	**Minute**						
			**0–15 ±SD**	**15–30 ±SD**	**30–45 ±SD**	**45–60 ±SD**	**60–75 ±SD**	**Number of average**	
1	Control^b^	0	280.4 ± 20.6	294.4 ± 4.3	311.4 ± 17.4	303.4 ± 14.7	297.4 ± 9.8	1,487.0	0

2	Lavender oil	0.1	217.6 ± 18.2	200.4 ± 12.4	195.6 ± 9.7	197.4 ± 7.9	193.2 ± 11.9	1,004.2	32.46

		0.3	187.8 ± 19.0	181.4 ± 20.5	186.2 ± 15.0	180.4 ± 16.5	178.4 ± 19.4	914.2	38.52

		0.5	139.6 ± 11.2	128.8 ± 10.9	116.8 ± 10.3	123.2 ± 8.9	118.2 ± 8.7	626.6	57.86

3	Nutmeg oil	0.1	120.0 ± 35.5	113.6 ± 32.9	114.2 ± 22.8	105.0 ± 22.3	100.2 ± 22.1	553.0	62.81

		0.3	121.4 ± 41.8	119.2 ± 37.1	110.8 ± 36.5	81.8 ± 23.2	82.2 ± 18.6	515.4	65.33

		0.5	107.8 ± 27.3	104.0 ± 26.1	84.4 ± 27.4	85.8 ± 23.1	84.6 ± 23.2	466.6	68.62

a SD: standard deviation;

b control: without essential oil;

c % Inhibitory effect = (Mean 1 − Mean 2/3)/Mean 1 ×100%.

**Table 3.** Active volatile compounds identified in blood after inhalation of essential oils of nutmeg seeds at different durations of inhalation.

**Compound**	**Duration of Inhalation**						**LRI Ref^a^**

	**½ hour (*R*^d^ = 84%)**		**1 hour (*R*^d^ = 90%)**		**2 hours (*R*^d^ = 86%)**		

	**LRI Exp^b^**	**Conc. μg/mL**	**LRI Exp^b^**	**Conc.** μ**g/mL**	**LRI Exp^b^**	**Conc. μg/mL**	
4-Terpineol	1181	1.5	1181	2.9	1183	6.3	1177
Safrole	nd	nd	nd	nd	1292	1.3	1285
Myristicin	1521	3.8	1521	5.2	1523	7.1	1520
Methyl myristate	1718	1.6	1719	1.4	1720	1.2	1726
Methyl palmitate	1919	67.8	1920	72.2	1922	58.7	1927
Palmitic acid	1952	2.8	nd	nd	nd	nd	1961**c**
Methyl octadeca-10-oate	2094	23.3	2096	24.7	2097	18.9	-
Methyl oleate	2100	12.1	2102	13.8	2105	10.7	-
Methyl stearate	2129	13.1	2132	13.2	2134	10.8	2128

Notes: nd = no detection;

a: LRI reference in Adams (1995) with DB5 column;

b: LRI experiment with DB5-MS column;

c: LRI reference in King *et al*. (1993) with HP5 column;

d: Recovery (*n* = 2) was calculated on the basis of comparison between 1,4-dichlorobenzene (methanol diluted) in blood plasma and 1,4-dichlorobenzene in methanol only.

**Table 4.** The average number of mice wheel cage rotations within 75 minutes of inhalation of myristicin, safrole, and 4-terpineol of nutmeg.

**Treatment**	**Dose (mL)**	**Mean Instead of Number of Average**							**Inhibitory Effect (%)**
		**Minutes**							
		**0–15**	**15–30**	**30–45**	**45–60**	**60–75**	**75–90**	**Total Number**	
**Control**	**Control**	321.5 ±30.1	305.64 ±13.3	299.89 ±10.2	287.96 ±18.2	314.6 ±35.6	255.46 ±25.34	1529.59	0
	**0.1 mL**	129.5 ±9.88	139.9 ±12.56	126.7 ±9.89	128.2 ±10.23	129.7 ±8.77	115.43 ±10.22	654	57.24
**Myristicin**	**0.3 mL**	105.8 ±19.10	120.4 ±20.5	129.5 ±15.08	117.6 ±16.5	109.2 ±19.4	76.45 ±3.20	582.5	61.92
	**0.5 mL**	55.23 ±11.2	60.8 ±10.9	45.4 ±10.3	39.2 ±8.9	8.33 ±8.7	0.81 ±0.01	208.96	86.34
	**0.1 mL**	120 ±35.5	113.6 ±32.9	114.2 ±22.8	105 ±22.3	100.2 ±22.1	98.43 ±15.67	553	63.85
**Safrole**	**0.3 mL**	95.4 ±11.12	91.34 ±22.23	83.26 ±16.34	90.45 ±22.22	88.2 ±13.16	45.29 ±10.23	448.65	70.67
	**0.5 mL**	40.2 ±7.2	49.5 ±6.14	30.55 ±7.35	18.6 ±6.78	14.7 ±8.2	0.00 ±0.00	153.55	89.96
	**0.1 mL**	132.22 ±33.55	133.21 ±23.4	124.98 ±22.8	125.65 ±32.3	105.2 ±22.1	111.56 ±32.32	621.26	59.38
**4-Terpineol**	**0.3 mL**	110.1 ±31.18	109.32 ±27.5	80.81 ±26.22	85.48 ±12.2	82.2 ±11.8	78.65 ±16.54	467.91	69.41
	**0.5 mL**	78.85 ±7.60	60.25 ±5.37	64.4 ±7.25	55.78 ±5.54	54.16 ±9.25	40.20 ±8.50	313.44	79.51
